# Safety and efficacy of ascending aorta cannulation during repair of acute type A aortic dissection (PA29-04): “Presented at the 65th Annual Scientific Meeting of the Japanese Association for Thoracic Surgery”

**DOI:** 10.1007/s11748-013-0355-9

**Published:** 2013-12-06

**Authors:** Masahiro Osumi, Hideichi Wada, Yuichi Morita, Masayuki Shimizu, Yuta Sukehiro, Mau Amako, Noritoshi Minematsu, Hitoshi Matsumura, Masaru Nishimi, Tadashi Tashiro

**Affiliations:** 1 Department of Cardiovascular Surgery, Fukuoka University Faculty of Medicine, Fukuoka, Japan; 2 7-45-1 Nanakuma, Jonan-ku, Fukuoka, 814-0133 Japan

**Keywords:** Aortic dissection, Ascending aorta perfusion, Ultrasound

## Abstract

**Objective:**

Antegrade central perfusion for acute Stanford type A aortic dissection prevents malperfusion and retrograde cerebral embolism during cardiopulmonary bypass. Prompt establishment of antegrade perfusion via the ascending aorta may improve surgical results of type A dissections, especially in situations of hemodynamic instability. Thus, we evaluated the safety and efficacy of cannulation of the dissected ascending aorta in acute type A dissection.

**Methods:**

We reviewed the medical charts of patients undergoing repair of acute ascending aortic dissection (*n* = 52) from April 2010 to April 2013. Cannulation was accomplished in 29 patients via the ascending aorta (central) and in 23 patients via the femoral or axillary artery (peripheral). The ascending aorta was routinely cannulated using Seldinger technique under epiaortic ultrasound guidance. Comorbidities, mortality, complications, and durations of hospital stays were compared for the groups.

**Results:**

In all cases, routine cannulation of the ascending aorta was safely performed with no resultant malperfusion or thromboembolism. Mean operative duration, cardiopulmonary bypass time, intubation time, and intensive care unit stay were significantly shorter in the central group. Two patients (6.8 %) in the central group died compared with four patients (17.3 %) in the peripheral group (*P* = 0.005).

**Conclusions:**

Antegrade central perfusion via the ascending aorta, a simple and safe technique that enables rapid establishment of antegrade systemic perfusion, was as safe as peripheral cannulation in patients with type A acute aortic dissection.

## Introduction

The optimal management of acute type A dissection of the ascending aorta is surgical intervention. Surgical results are influenced by perfusion technique and cannulation site for cardiopulmonary bypass (CPB), of which 3 are currently used. Cannulation using the common femoral artery is the standard option but can carry some risk of critical organ malperfusion or retrograde embolization. Axillary cannulation, which requires sewing a graft or repairing the axillary artery, has recently become a widely used approach for arterial cannulation. However, in patients of small stature, a concern may be shortage of pump flow due to a narrow axillary artery. Finally, ascending aorta cannulation, which may be suitable for rapid establishment of CPB in cases of hemodynamic instability, has been used occasionally.

## Subjects and methods

Between April 2010 and April 2013, 52 patients with Stanford type A acute aortic dissection (mean age 69.6; 20 men and 32 women) were treated surgically, either urgently or emergently, with hemiarch or arch replacement under deep hypothermic circulatory arrest.

All patients had a computed tomographic angiogram for diagnosis and operative planning, and the cannulation site was established before going to the operating room. Although the sites of cannulation varied, the approaches to cooling and circulatory arrest were similar. The goal of repair was prevention of proximal rupture and preservation of aortic valve competence.

The arterial cannulation site was decided individually, according to patient status and, more dominantly, by surgeon preference. In cases with aortic cannulation, an undissected site of the lesser curve in the aortic arch was usually chosen for cannulation. If the aorta was circularly dissected or nearly complete 360° dissection of the aorta occurs, resulting in an essentially free-floating ascending aortic true lumen, we choose the femoral or axillary artery for the cannulation site. In cases with hemodynamic instability, we try to choose femoral arterial cannulation. We try to avoid femoral arterial cannulation in elderly patients with extensive aortic atheroma on preoperative imaging to avoid potential retrograde embolization.

**Fig. 1:**
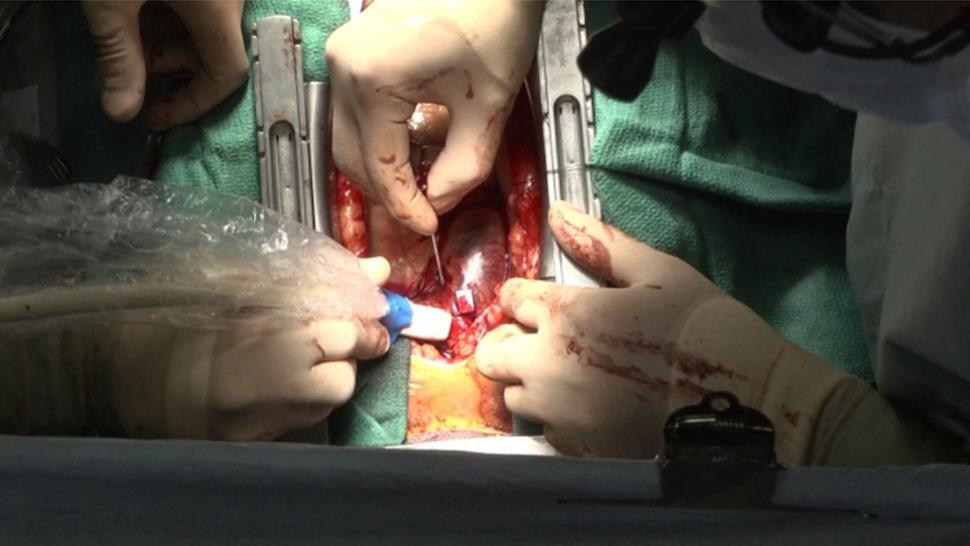
A single pledgeted 4–0 polypropylene mattress suture was placed at the left lateral aspect of the ascending aorta

**Fig. 2:**
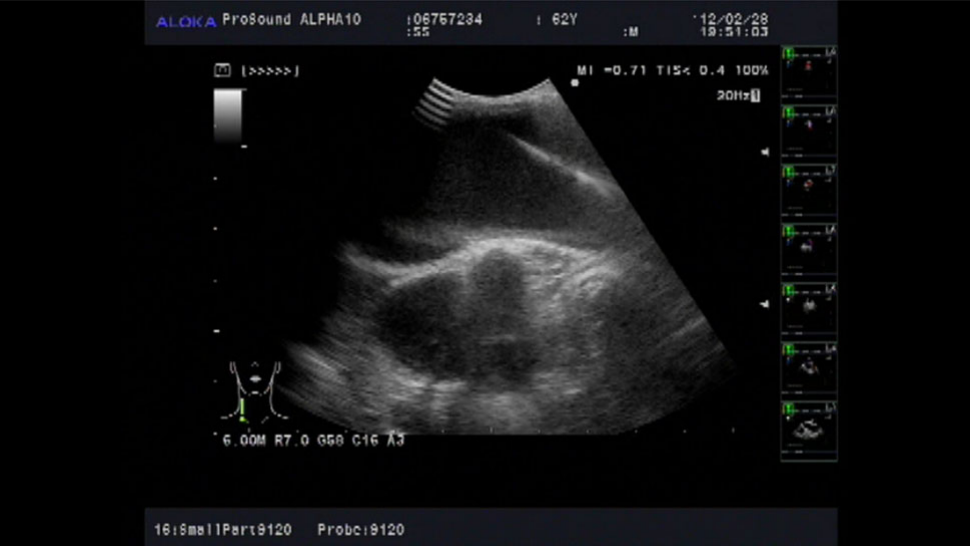
To ensure correct positioning of the tip of the arterial cannula in the true lumen by epiaortic ultrasound guidance

**Fig. 3:**
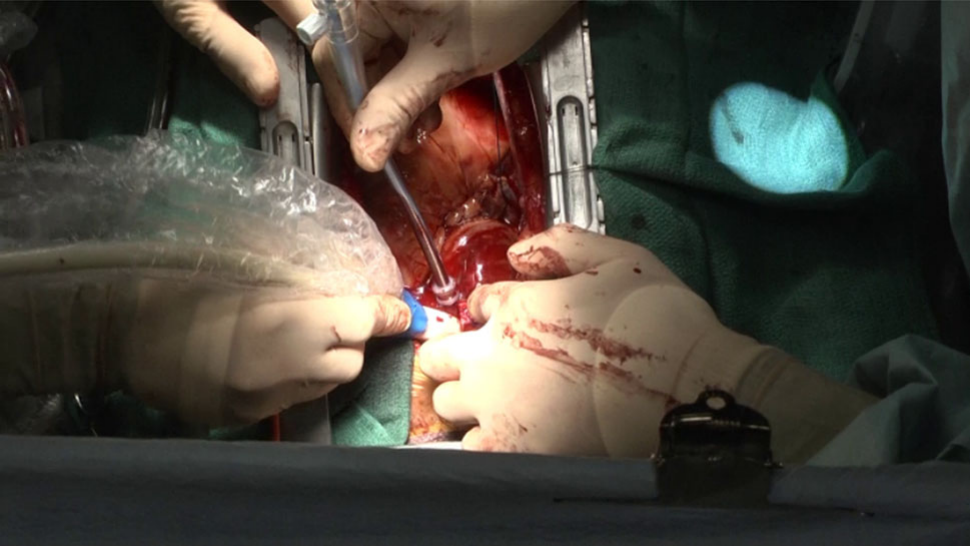
A 16-French, heparin-coated, flexible, thin-walled cannula (Fem-Flex II Femoral Arterial Cannulae; Edwards Life Sciences LLC, Midvale, UT, USA) was inserted into the aorta using Seldinger technique

Cases involving central cannulation (*n* = 29) were performed through a median sternotomy. A single pledgeted 4–0 polypropylene mattress suture was placed on the ascending aorta, and a 16-French, heparin-coated, flexible, thin-walled cannula (Fem-Flex II Femoral Arterial Cannulae; Edwards Life Sciences LLC, Midvale, UT, USA) was inserted into the aorta using Seldinger technique and antegrade systemic perfusion was performed, as shown in Fig. 1. We used transesophageal and epiaortic echocardiography to ensure correct positioning of the tip of the arterial cannula in the true lumen and color Doppler imaging to assess perfusion flow, as shown in Figs. 2 and 3. Venous cannulae were inserted into the superior and inferior vena cava. A left ventricular vent cannula was inserted through the right superior pulmonary vein, and systemic cooling was started immediately. Myocardial protection was obtained by means of retrograde infusion of cold blood cardioplegia. If an intimal tear was found in the ascending aorta or proximal transverse aorta, replacement of the ascending aorta or hemiarch was performed using an open distal anastomosis under circulatory arrest with retrograde cerebral perfusion. In cases in which an intimal tear was found in the transverse aorta, total arch replacement was performed to exclude intimal tear. We use selective antegrade cerebral perfusion whenever total arch replacement is required.

Patients undergoing femoral artery cannulation (*n* = 7) underwent femoral cutdown. A purse-string suture was placed on the anterior surface of the femoral artery, and a cannula was placed through the purse-string suture using Seldinger technique.

Patients undergoing axillary cannulation (*n* = 16) underwent axillary cutdown. The arteriotomy was started with a knife and completed with an aortic punch, and an 8-mm Dacron graft was sewn to the hole that was created.

## Results

Preoperative comorbidities were similar for the 2 groups, as shown in Tables 1 and 2. Cannulation of the dissected ascending aorta with systemic antegrade perfusion was successfully performed in all cases, and in no case was switching of the cannulation site required. Neither malperfusion nor thromboembolism occurred as a result of ascending aorta cannulation.

**Table 1:** Preoperative patients’ characteristics

Variable	Central group (*n* = 29)	Peripheral group (*n* = 23)	*P* value
Age (mean ± SD)	71.7 ± 10.4	67.1 ± 11.1	0.34
Female gender	19 (29.4 %)	13 (69.5 %)	0.001
Hypertension	24 (70.5 %)	14 (60.8 %)	0.15
Diabetes mellitus	4 (11.7 %)	1 (6.7 %)	0.41
COPD	1 (2.9 %)	1 (4.3 %)	0.62
Renal dysfunction (Cre >1.5)	2 (5.8 %)	2 (8.6 %)	0.83
Previous stroke	6 (17.6 %)	5 (21.7 %)	0.65

**Table 2:** Dissection-related complications

Variable	Central group, *n* = 29 (%)	Peripheral group, *n* = 23 (%)	*P* value
Pericardiac effusion	15 (51.7)	13 (56.5)	0.53
Aortic valve regurgitation	3 (10.3)	4 (17.3)	0.61
Cardiac tamponade	3 (10.3)	2 (8.6)	0.82
Shock	3 (10.3)	2 (8.6)	0.74
Myocardial ischemia	1 (3.4)	1 (4.3)	0.82
Cerebral ischemia	5 (20.6)	6 (26.0)	0.48
Limb ischemia	0 (0)	1 (4.3)	0.44

Preoperative and postoperative data are summarized in Tables 3 and 4. The central group, which was cannulated via the ascending aorta, consisted of the 25 patients with replacement of the aortic hemiarch, and the 4 patients with total arch replacement. The peripheral group, which received axillary or femoral cannulation, consisted of the 16 patients with aortic hemiarch replacement, including 1 concomitant cardiac arterial bypass graft, and the 7 patients with total arch replacement, with 1 receiving concomitant aortic valve replacement. The mean operation time (381.4 vs. 512.5 min; *P* < 0.001) and mean CPB time (209.4 vs. 232.9 min; *P* < 0.001) and the interval time between the start of operation and the start of CPB (44.4 vs. 98.4 min; *P* < 0.001) were significantly shorter in the central group.

**Table 3:** Operative data

	Central group (*n* = 29)	Peripheral group (*n* = 23)	*P* value
Hemiarch replacement	25 (86.2 %)	16 (69.5 %)	0.36
Total arch replacement	4 (13.7 %)	7 (30.4 %)	0.21
Concomitant procedure	0 (0 %)	2 (2.1 %) 1; AVR 1; CABG	0.22
Operative time (min)	381.4	512.5	<0.001
CPB time (min)	209.4	232.9	<0.001

**Table 4:** Postoperative data

	Central group (*n* = 29)	Peripheral group (*n* = 23)	*P* value
Reoperation for bleeding	1 (3.4 %)	1 (4.7 %)	0.29
Deep sternal infection	1 (3.4 %)	1 (4.7 %)	0.64
Permanent stroke	2 (6.8 %)	3 (13.0 %)	0.24
Respiratory failure	3 (10.2 %)	3 (13.0 %)	0.41
Intubation time (h)	90.8 ± 71.2	80.8 ± 61.2	0.03
Mortality (30 days)	2 (6.8 %)	4 (17.3 %)	<0.001

There were no significant intergroup differences in the frequency of hemorrhage requiring redo thoracotomy, which occurred in 1 patient (3.4 %) in the central group and in 1 patient (4.3 %) in the peripheral group. The mean intubation time (90.8 vs. 80.8 h) was not different statistically, but a difference is recognized between the 2 groups.

Two patients (6.8 %) in the central group died from multisystem organ failure compared with 4 patients (17.3 %) in the peripheral group (*P* < 0.001); 2 from multisystem organ failure, 1 from heart failure, and 1 from renal failure.

## Discussion

The optimal cannulation site for the repair of acute type A dissection is still not known. The most popular site for cannulation in this setting was the femoral artery until the late 1990s [1]. We believe that ascending aortic cannulation in the repair of acute type A dissection is technically feasible, safe, and an effective alternative to traditional methods of axillary or femoral cannulation. The femoral artery is the most common cannulation site that is easily accessible at the same time as median thoracotomy. Retrograde perfusion from the femoral artery is associated with the risk of the elevation of a dissected intimal flap, expansion of the false lumen, and retrograde embolization due to thrombus or atherosclerotic debris [2–4]. Axillary artery cannulation may have advantages over femoral artery cannulation in preventing organ malperfusion [5–7]. However, axillary artery cannulation also presents certain problems as it requires a more precise technique and more time [6]. If the subclavian artery is involved in the dissection, cannulation of the axillary artery may cause retrograde carotid dissection and cerebral malperfusion [8]. Moreover, axillary artery cannulation may cause local complications, including injury of the artery or the brachial plexus and arm ischemia, often resulting in insufficient CPB flow due to the small size of the artery, and atherosclerosis of the artery. The axillary artery may not be suitable for rapid and secure establishment of antegrade perfusion.

One of the major complications associated with type A dissection is organ malperfusion [9–11]. We propose two preventative measures for this: one is reliable true lumen perfusion and the other is antegrade natural blood flow. Retrograde perfusion from the femoral artery is associated with risks of elevation of a dissected intimal flap, expansion of the false lumen, and retrograde embolization due to thrombus or atherosclerotic debris [3, 4, 12].

In the present study, central cannulation using our method was technically feasible and safe in all patients evaluated. With careful and safe cannulation technique, we encountered no difficulties related to the cannulation procedure and did not require switching to another cannulation site. If the non-dissected area was adequate and there was no risk of embolus associated with cannulation through the thrombosed false lumen, central cannulation was performed [13]. We began introducing this technique in 2011 for type A aortic dissection and have safely performed it on all 20 of our most recent cases.

The technical concerns regarding this ascending cannulation technique are danger of aortic rupture at the cannulation site and cannulation into the false lumen. In regard of the first issue, Khaladj et al. [14] reported only 1 of 122 patients (0.8 %) who had aortic rupture caused by aortic cannulation in patients with acute type A dissection. We saw no aortic rupture after aortic cannulation. Kamiya et al. [1] reported that the false lumen cannulation can be recognized after cross-clamping of the ascending aorta through a pressure difference of the radial arteries or a sudden perfusion pressure elevation in the arterial cannula. In such cases, the aorta was opened for a brief period of circulatory arrest: an incision was made in the membrane between the true and false lumens; the cannula was repositioned; and cardiopulmonary bypass was reestablished. This bail-out technique was used in 4 patients in their series, and the problem of false lumen cannulation was solved without malperfusion [1, 14–16]. We have no such case. However, the safety of this procedure may be represented by the reliability of true lumen perfusion via ascending aorta. Routine application of epiaortic ultrasonographic guidance combined with Seldinger technique could eliminate false lumen cannulation.

As a result, we consider that the reason is that central group has a shorter operative and CPB time than peripheral group, which being few concomitant procedure and total arch replacement. Therefore, we infer that the improvement results of central group has connected a shorter operative and CPB time.

## Conclusion

In conclusion, our study has shown that direct central cannulation through the ascending aorta was successful for repair of type A dissection and produced surgical results superior to those of peripheral cannulation. Central cannulation is a safe and useful technique that enables rapid establishment of antegrade systemic perfusion in select patients with acute type A aortic dissection.
